# Kinetics and Energetics of Thermal *Cis-Trans* Isomerization of a Resonance-Activated Azobenzene in BMIM-Based Ionic Liquids for PF_6_^−^/Tf_2_N^−^ Comparison

**DOI:** 10.3390/molecules22081273

**Published:** 2017-07-29

**Authors:** Guido Angelini, Cristina Campestre, Luca Scotti, Carla Gasbarri

**Affiliations:** 1 Department of Pharmacy, University “G. d’Annunzio” of Chieti-Pescara, via dei Vestini, 66100 Chieti, Italy; guido.angelini@unich.it (G.A.); cristina.campestre@unich.it (C.C.); 2 Department of Oral Science, Nano and Biotechnology, University “G. d’Annunzio” of Chieti-Pescara, via dei Vestini, 66100 Chieti, Italy; l.scotti@unich.it

**Keywords:** imidazolium ionic liquids, 4-methoxyazobenzene, rotation, Arrhenius plot, Eyring plot

## Abstract

BMIM PF_6_ (1-butyl-3-methylimidazolium hexafluorophosphate) and BMIM Tf_2_N (1-butyl-3-methylimidazolium bis(trifluoromethylsulfonyl)imide) are two *conventional* room-temperature ionic liquids widely employed and investigated as reaction media. Despite the presence of the same imidazolium ring in their structure they are different in many chemical and physical properties due to the nature of the anions. The thermal *cis-trans* isomerization of an electronically activated azobenzene have been used as reaction model to compare the behavior of PF_6_^−^ and Tf_2_N^−^. Rotation is the mechanism by which the investigated azobenzene is converted into the *trans* isomer spontaneously in the dark both in BMIM PF_6_ and in BMIM Tf_2_N. The kinetic rate constants of the process have been determined at different temperatures and the activation energies of the reaction have been calculated according to the Arrhenius and Eyring equations. The results presented herein highlight different solute-solvent interactions involving the PF_6_^−^ and Tf_2_N^−^ anions during the *cis-trans* isomerization.

## 1. Introduction

Despite the existence of a large variety of photoreactive compounds and light-switchable devices, azobenzene and its derivatives still represent relevant molecules due to their spectroscopic properties and isomerization mechanisms. External stimuli are requested to induce the *trans-cis* photoisomerization [1,2,3], while the thermal *cis-trans* conversion occurs spontaneously in the dark owing to the fact that the *trans* is about 40–50 kJ/mol more stable than the *cis* isomer [4,5].

Rotation and inversion are the competitive mechanisms proposed for the *cis-trans* isomerization: the former takes place by the formation of a dipolar transition state in which the nitrogen-nitrogen π-bond is heterolytically broken, while the latter involves the rehybridation of one of the nitrogen atoms from *sp*^2^ to *sp* and the formation of a linear transition state in which the double bond is retained [6]. Solvent polarity and viscosity, as well as the steric and electronic effects of a substituent, can promote one mechanism over the other [7]. Typical V-shaped Hammett plots have been obtained in different media for the *cis-trans* isomerization of monosubstitued azobenzenes confirming that rotation is favored by an electron-donating group, while inversion is associated to an electron-withdrawing substituent [8,9,10]. Recently, V-shaped Hammett plots have been obtained in high polar ionic liquids, as BMIM Tf_2_N, while linear plots have been observed in high viscous ionic liquids, as BMIM PF_6_, suggesting a change of mechanism, from rotation to inversion, in the presence of electron-withdrawing substituents [11]. Since the reaction of monosubstitued azobenzenes occurs by rotation in a polar environment and by inversion in nonpolar media, the behavior of BMIM PF_6_ resembles that of a polar solvent in comparison to BMIM Tf_2_N in disagreement with the relative dielectric constant values [12]. Generally, BMIM-based ionic liquids are characterized by ordered structural networks in which the hydrogen atoms in position 2, 4 and 5 of the imidazolium ring and the hydrogens of the *N*-alkyl groups are involved in hydrogen bonds. Moreover, one imidazolium cation is connected by three anions and each anion is surrounded by three imidazolium cations [13]. It has been widely demonstrated the fundamental role of the anion for the physicochemical properties of BMIM-based ionic liquids [14,15,16,17,18,19]. In particular, many kinds of reactions have been carried out in BMIM PF_6_ and BMIM Tf_2_N [20,21,22]. In addition to viscosity and polarity, they show different surface tension, density, conductivity, molecular weight, thermal behavior, polarizability and dipolarity [23,24,25,26,27,28,29]. The aim of this work has been the investigation of the kinetic rate constants and the energetic parameters associated with the thermal isomerization of 4-CH_3_O-azobenzene (MeO-AB) in BMIM PF_6_ and BMIM Tf_2_N to compare the effect of PF_6_^−^ and Tf_2_N^−^ and contribute to the characterization of the two ionic liquids for their application as reaction media. The presence of the methoxy group in the *para* position strongly increases the electron density in the π* orbital of the MeO-AB molecule by positive mesomeric effect leading to an highly dipolar transition state without inducing strong steric effects which generally hinder rotation [30,31]. Although closer energetic barriers have been observed for the *cis-trans* conversion of MeO-AB and unsubstitued azobenzene and despite of the inversion represents the dominant mechanism in the case of azobenzene, the thermal isomerization of the MeO-AB molecule proceeds only through the rotation pathway both in organic solvents and ionic liquids [32,33] (Supplementary Materials, A–B).

## 2. Results

### 2.1. Thermal Cis-Trans Isomerization

**Figure 1:**
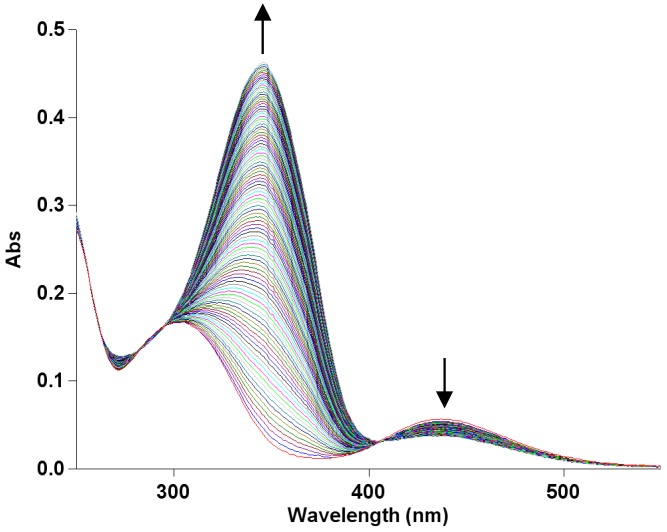
UV-vis spectra for the thermal *cis-trans* isomerization of MeO-AB in BMIM PF_6_ at 40 °C.

After irradiation, the MeO-AB solution shows a shift in the maximum absorption band from 347 to 303 nm and the presence of a broad band at 440 nm in both the investigated ionic liquids confirming the conversion of the *trans* into the *cis* isomer. In the dark, the thermal *cis-trans* isomerization occurs and is carried out by the appearance of three isosbestic points at 258, 295 and 407 nm. The UV-vis spectra of the *cis-trans* conversion of MeO-AB in BMIM PF_6_ at 40 °C are reported in Figure 1 as an example. 

The first-order rate constants (*k*_obs_) measured in BMIM PF_6_ and BMIM Tf_2_N at different temperatures are reported in Table 1.

### 2.2. Arrhenius and Eyring parameters

**Figure 2:**
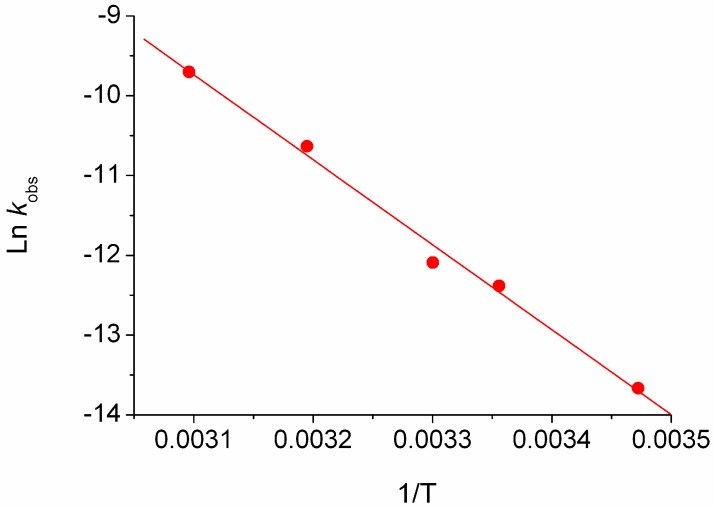
Arrhenius plot of MeO-AB in BMIM Tf_2_N.

The energetic parameters of the reaction have been estimated by applying the temperature dependent Arrhenius and Eyring equations as described in the Experimental Section. The Arrhenius plot shows a good correlation in both the ionic liquids. The linear fit obtained for the BMIM Tf_2_N is shown in Figure 2 as an example.

**Figure 3:**
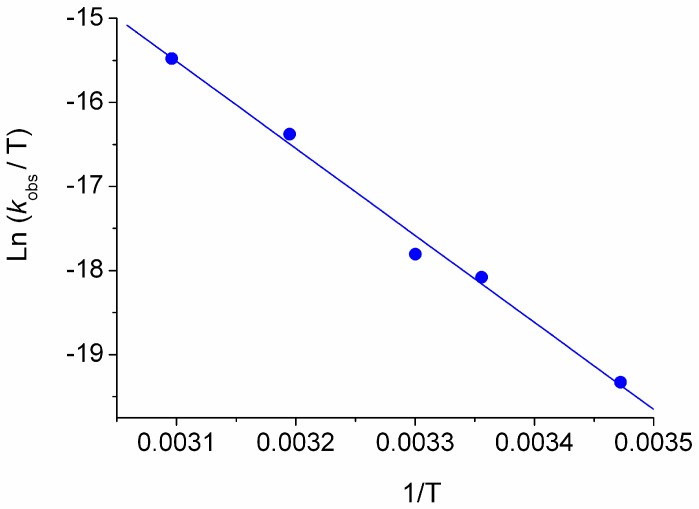
Eyring plot of MeO-AB in BMIM Tf_2_N.

Similarly, linear plots have been determined by applying the Eyring equation in the investigated ionic liquids. The Eyring plot obtained for the BMIM Tf_2_N is reported in Figure 3 as an example.

The energetic parameters of the *cis-trans* isomerization of MeO-AB calculated from the Arrhenius and Eyring plots are shown in Table 2.

## 3. Discussion

Generally, temperature has no effect on the spectroscopic behavior of azobenzene and derivatives, but strongly enhances the isomerization rate [34]. The thermal isomerization of MeO-AB becomes faster by increasing temperature in both the ionic liquids and higher *k*_obs_ values are obtained at higher temperature. In particular, the *k*_obs_ increases of about 108 times in BMIM PF_6_ and 52.5 times in BMIM Tf_2_N passing from 288 to 323 K.

The obtained E_a_ values are similar to those observed for the *cis-trans* conversion of azobenzene in conventional solvents, corresponding to 84–104 kJ/mol [35]. Moreover, these data are in agreement with the activation energy and frequency factor reported for 3-butyl-1-methyl-2-phenylazoimidazolium, corresponding to 85 ± 4 kJ/mol and (1.8 ± 1.7) × 10^11^ s^−1^ in BMIM PF_6_ and 85 ± 3 kJ/mol and (1.3 ± 1.0) × 10^11^ s^−1^ in BMIM Tf_2_N [36]. Although in BMIM PF_6_ the kinetic rate of the MeO-AB isomerization is faster in comparison to BMIM Tf_2_N in the investigated temperature range (Table 1), the E_a_ and ΔH^≠^ values are higher for BMIM PF_6_ in comparison to BMIM Tf_2_N (Table 2). Both E_a_ and ΔH^≠^ correspond to the barrier height required to activate the reagent molecules from their initial state to the transition state of a reaction, and for the thermal *cis-trans* isomerization the initial state consists in the solvation of the *cis* isomer by the reaction medium as previously described by Asano et al. [33]. In the presence of an electron-donor monosubstitued azobenzene the barrier height of the reaction depends on the solute-solvent interactions and tends to decrease by increasing the solvent polarity [34]. The MeO-AB molecule is solubilized in the *trans* form in the ionic liquids, then the solutions are irradiated by UV-visible light to induce the conversion from *trans* to *cis* isomer. The strong change in the molecular geometry of the MeO-AB reduces the distance between the 4-4’positions of the azobenzene moiety and consequently increases the dipole moment from 1.86 to 4.54 D [8]. The lower E_a_ and ΔH^≠^ obtained for BMIM Tf_2_N indicate that the more polar ionic liquids provide a more efficient stabilization of the *cis* MeO-AB in the ground state in comparison to BMIM PF_6_ [37] without changing the energy associated to the transition state [34] as suggested by the higher frequency factor A and the activation entropy ΔS^≠^ (Table 2). On the other hand, the activation entropy ΔS^≠^ calculated according to the Eyring equation is referred to the redistribution of the energy of the molecule in the transition state to allow the rotation pathway. The obtained ΔS^≠^ values indicate that BMIM PF_6_ offers the best stabilization of the dipolar transition state of the reaction in comparison to BMIM Tf_2_N. In particular, the largest ΔS^≠^ implies the largest frequency factor A and reflects the faster isomerization rates [32] in agreement with the data obtained for the investigated ionic liquids. This behavior can be attributed to the specific properties of the PF_6_^−^ in comparison to Tf_2_N^−^, including smaller van der Waals radius, higher mobility and lower dispersion charge, involved in the solvation of the dipolar transition state in which the formal charges generated by the resonance effect of the methoxy group are stabilized by strictly oriented anions and cations [33]. 

## 4. Materials and Methods

### 4.1. Materials and Instruments

BMIM PF_6_ (1-butyl-3-methylimidazolium hexafluorophosphate) and BMIM Tf_2_N (1-butyl-3-methylimidazolium bis(trifluoromethylsulfonyl)imide) were purchased from IoLiTec and stirred under vacuum at 60 °C overnight prior to use to decrease the water content below the limit detectable by spectroscopic techniques [28,38]. 4-CH_3_O-azobenzene (MeO-AB) was synthetised as previously described [8]; ethanol (99% spectroscopy grade) was purchased from Fluka and used without further purification. The irradiation to induce the *trans-cis* isomerization was performed by means of a Hg−Xe arc lamp (150 W) equipped with a band-pass interference filter centered at 365.0 +2/−0 nm wavelength and 10.0 +2/−2 nm bandwidth. The UV-vis spectra for the thermal *cis-trans* isomerization were recorded by using a Cary 1-E spectrophotometer. 

### 4.2. Methods

A 2.6 × 10^−3^ M ethanolic solution of MeO-AB was prepared and kept in the dark at room temperature at least for 4 days before use. An appropriate amount was transferred into a 1 cm light path quartz cuvette containing 200 μL of BMIM PF_6_ or BMIM Tf_2_N to obtain a final concentration of 2.6 × 10^−5^ M. The sample was dried under Nitrogen flow to remove the organic solvent [39,40] and then was irradiated for 45 min. The decreasing of the high-intensity absorption band at 347 nm (due to the π → π* transition) and the increasing of the low-intensity band at about 440 nm (due to the n → π* transition) were used as evidence for the *trans-cis* photoisomerization. The thermal *cis-trans* conversion follows a first order decay [41,42,43,44] and the kinetic rate constants (*k*_obs_) were spectrophotometrically measured in the temperature range 15−50 (± 0.1)°C by monitoring the absorption change at the maximum wavelength of the trans isomer in the dark over a period of about 24 h (Supplementary Materials C–D) as previously described [8]. 

The activation energy (E_a_) and frequency factor (A) have been calculated from the *k*_obs_ values by the Arrhenius equation (1).

ln *k*_obs_ = ln A − E_a_/RT, (1)

where R is the universal gas constant and T is the absolute temperature. 

The activation enthalpy (ΔH^≠^) and the activation entropy (ΔS^≠^) have been calculated by the Eyring equation (2).

ln (*k*_obs_/T) = −ΔH^≠^/RT + ln (*k*_B_/*h*) + ΔS^≠^/R, (2)

where *k*_B_ and *h* are the Bolzmann and Planck constants, respectively.

## 5. Conclusions

The thermal isomerization of the investigated MeO-AB represents a model reaction to understand the behavior of BMIM-based ionic liquids. The *cis-trans* conversion occurs by rotation in BMIM PF_6_ and BMIM Tf_2_N according to the electronic effect and the position of the methoxy group in the azobenzene molecule. The results presented herein indicate that best solvation of the *cis* isomer in the ground state occurs in the presence of the Tf_2_N^−^ anions as suggested by the activation energy and the activation enthalpy, while the reaction is faster in the presence of the PF_6_^−^ anions as pointed out by the kinetic rate constants, the frequency factor and the activation entropy. The different behavior shown by the investigated ionic liquids can be attributed to the stronger solvation capability of the *cis* isomer of MeO-AB in the ground state by BMIM Tf_2_N and the stronger stabilization of the dipolar transition state by BMIM PF_6_.

## Figures and Tables

**Table 1:** First-order rate constants for the thermal isomerization of MeO-AB in the investigated ionic liquids.

T (K)	BMIM PF_6_ 10^−6^ *k*_obs_/s^−1^	BMIM Tf_2_N 10^−6^ *k*_obs_/s^−1^
288 ± 0.1	1.19 ± 0.1	1.16 ± 0.2
298 ± 0.1 ^1^	6.83 ± 0.1	4.19 ± 0.7
303 ± 0.1	8.42 ± 0.1	5.61 ± 0.1
313 ± 0.1	25.5 ± 0.2	24.1 ± 0.1
323 ± 0.1	129.6 ± 0.1	61.2 ± 0.1

^1^ Reference [11].

**Table 2:** Activation energy E_a_, frequency factor A, activation enthalpy ΔH^≠^ and activation entropy ΔS^≠^ for the thermal isomerization of MeO-AB in BMIM PF_6_ and BMIM Tf_2_N.

Ionic Liquid	E_a_ (kJ/mol)	A (s^−1^)	ΔH^≠^ (kJ/mol)	ΔS^≠^ (J/K mol)
BMIM PF_6_	98.4 ± 3.1	(8.93 ± 2.5) × 10^11^	95.9 ± 3.0	70.3 ± 1.9
BMIM Tf_2_N	88.6 ± 2.8	(0.13 ± 0.1) × 10^11^	86.0 ± 2.6	35.2 ± 1.1

## Supplementary Materials

Supplementary materials are available online.
