# Supplementary material for: Kinetics and Energetics of Thermal Cis-Trans Isomerization of a Resonance-Activated Azobenzene in BMIM-Based Ionic Liquids for PF6−/Tf2N− Comparison
Source: Molecules. 2017 Jul 29;22(8):1273. doi: 10.3390/molecules22081273 (PMC6152290; doi:10.3390/molecules22081273)
Supplement: Supplementary file 1 [file molecules-22-01273-s001.pdf]

- Electronic Supplementary Materials -

Kinetics and Energetics of Thermal Cis-Trans Isomerization of a Resonance-Activated Azobenzene in BMIM-based ionic liquids for PF_6^- / Tf_2N^- Comparison

Guido Angelini[1], Cristina Campestre[1], Luca Scotti[2], Carla Gasbarri*[,1]

[1] Department of Pharmacy, University "G.d'Annunzio" of Chieti-Pescara, via dei Vestini, 66100 Chieti, Italy

[2] Department of Oral Science, Nano and Biotechnology, University "G.d'Annunzio" of Chieti-Pescara, via dei Vestini, 66100, Chieti, Italy

*Corresponding author. carla.gasbarri@unich.it; +39-0871-3554786

Index

A. Scheme of the thermal cis-trans isomerization of azobenzene by rotation

B. Chemical structures of the investigated molecules

C. 4-MeO-azobenzene in BMIM Tf_2N

Examples of UV-vis spectra in the temperature range 15-50°C before irradiation.

Examples of the absorbance changes after the irradiation during the time for each temperature.

D. 4-MeO-azobenzene in BMIM PF_6

Examples of UV-vis spectra in the temperature range 15-50°C before irradiation.

Examples of the absorbance changes after the irradiation during the time for each temperature.

A. Scheme of the thermal cis-trans isomerization of azobenzene by rotation

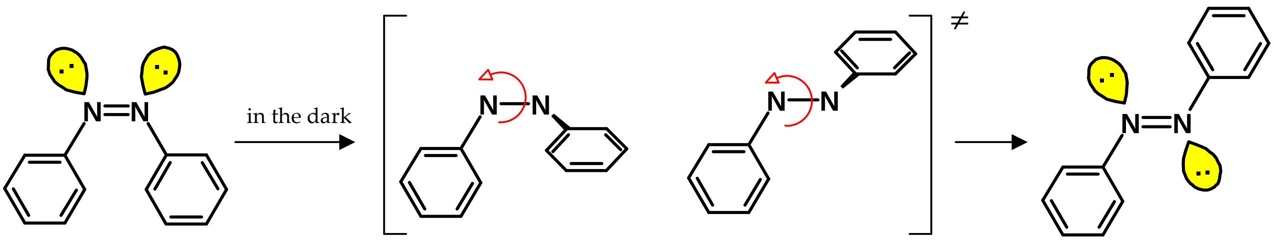

Cis Azobenzene

Trans Azobenzene

B. Chemical structures of the investigated molecules

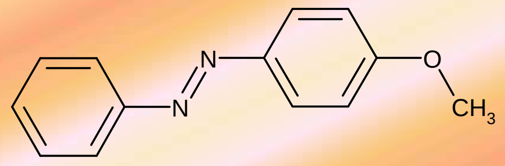

4-MeO-azobenzene

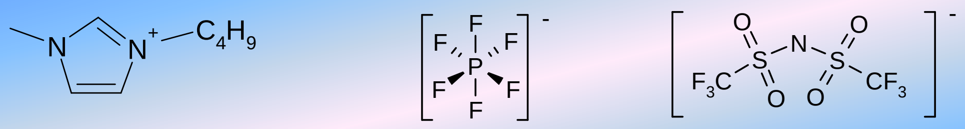

BMIM

PF^-_6

Tf_2N^-

C. 4-MeO-azobenzene in BMIM Tf_2N

1) 15°C UV-vis spectrum before irradiation

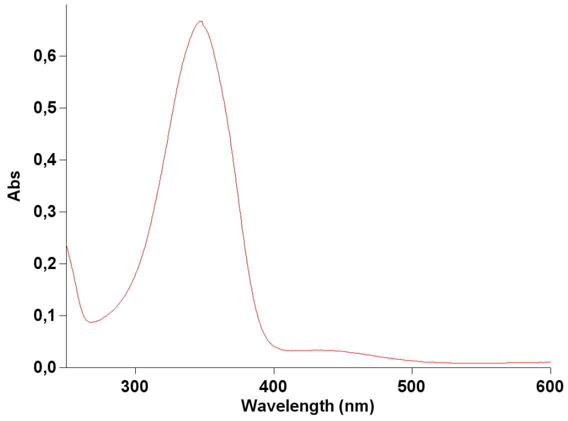

Time (s)	Abs after irradiation
0	0.0417
3600	0.04271
7200	0.04554
10800	0.04826
14400	0.0509
18000	0.05342
21600	0.05635
25200	0.05882
28800	0.06111
32400	0.06323
36000	0.06568
39600	0.06823
43200	0.07097
46800	0.0735
50400	0.07596
54000	0.07829
57600	0.08072
61200	0.08336
64800	0.08559
68400	0.088
72000	0.0908
75600	0.09289

2) 25°C UV-vis spectrum before irradiation

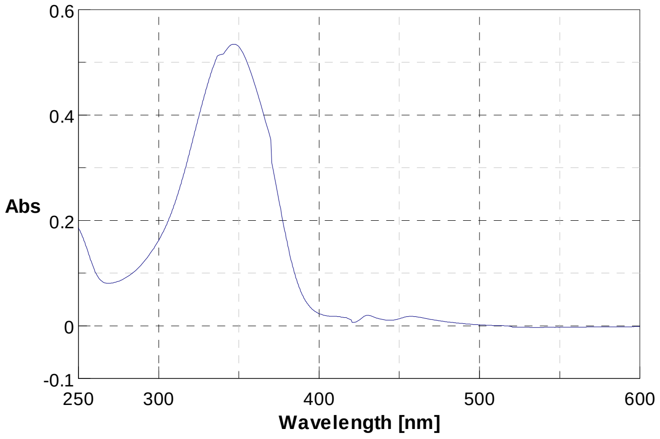

Time (s)	Abs after irradiation
0	0.02749
3600	0.03541
7200	0.04202
10800	0.04848
14400	0.05499
18000	0.06113
21600	0.06718
25200	0.07307
28800	0.07881
32400	0.08439
36000	0.09
39600	0.09546
43200	0.10077
46800	0.10599
50400	0.11114
54000	0.11643
57600	0.12137
61200	0.12646
64800	0.13123

3) 30°C UV-vis spectrum before irradiation

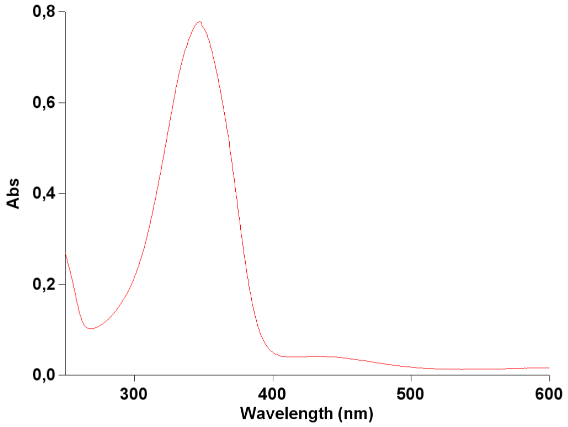

Time (s)	Abs after irradiation
0	0.047
1800	0.055
3600	0.06324
5400	0.07092
7200	0.07843
10800	0.08563
14400	0.09955
18000	0.1135
21600	0.1271
25200	0.1402
28800	0.153
32400	0.1655
36000	0.1782
39600	0.1903
43200	0.2021
46800	0.214
50400	0.2256
54000	0.2366
57600	0.2479

4) 40°C UV-vis spectrum before irradiation

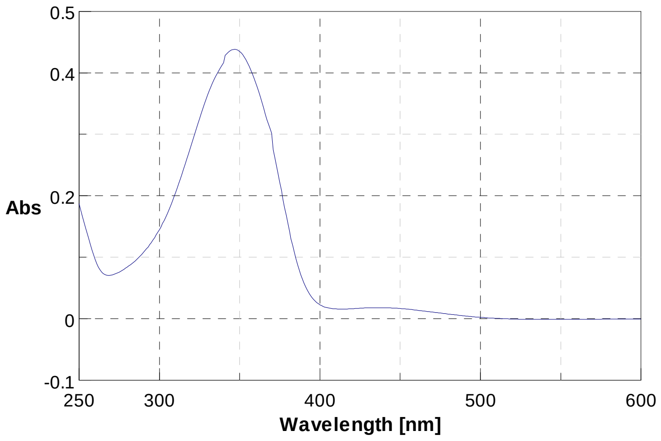

Time (s)	Abs after irradiation
0	0.02694
600	0.03362
1200	0.03956
1800	0.04546
2400	0.05119
3000	0.05715
3600	0.06229
4200	0.0677
4800	0.07268
5400	0.07797
6000	0.08291
6600	0.08804
7200	0.09312
7800	0.09806
8400	0.10736
9000	0.11207
9600	0.1165
10200	0.12097
10800	0.12553
11400	0.13002
12000	0.13425
12600	0.13836
13200	0.1423
13800	0.1467
14400	0.1507
15000	0.1548
15600	0.1585
16200	0.1624
16800	0.16637
17400	0.17015
18000	0.17389
18600	0.17754
19200	0.1809
19800	0.185
20400	0.1888

5) 50°C UV-vis spectrum before irradiation

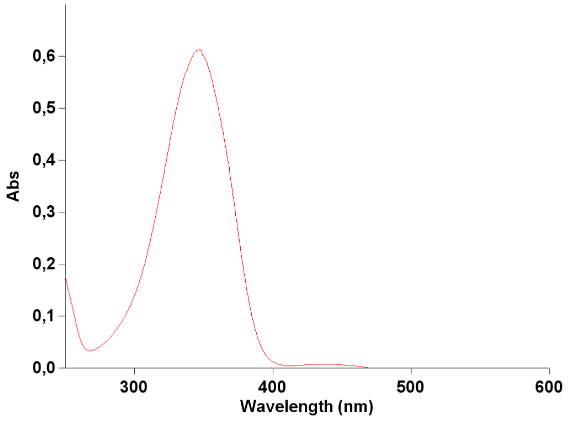

Time (s)	Abs after irradiation
0	0.01412
300	0.02175
600	0.03263
900	0.04365
1200	0.05471
1500	0.06493
1800	0.07468
2100	0.08428
2400	0.09368
2700	0.1031
3000	0.1123
3300	0.1215
3600	0.1304
3900	0.1389
4200	0.1477
4500	0.1558
4800	0.164
5100	0.1724
5400	0.1805
5700	0.188
6000	0.1957
6300	0.2032
6600	0.2109
6900	0.218
7200	0.225
7500	0.2319
7800	0.2386
8100	0.2455
8400	0.2521
8700	0.259
9000	0.2653
9300	0.2713
9600	0.2774
9900	0.2834
10200	0.2897

D. 4-MeO-azobenzene in BMIM PF_6

1) 15°C UV-vis spectrum before irradiation

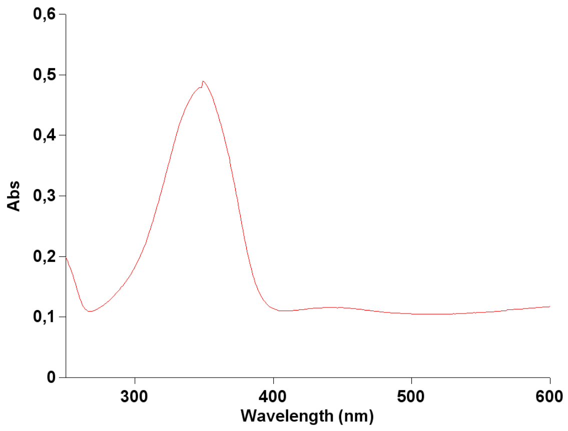

Time (s)	Abs after irradiation
0	0.00395
3600	0.00693
7200	0.00913
10800	0.01072
14400	0.01224
18000	0.014
21600	0.01582
25200	0.01753
28800	0.01989
32400	0.02116
36000	0.02369
39600	0.02599
43200	0.02777
46800	0.02991
50400	0.03194
54000	0.0338
57600	0.03665
61200	0.03811
64800	0.04044
68400	0.04269

2) 25°C UV-vis spectrum before irradiation

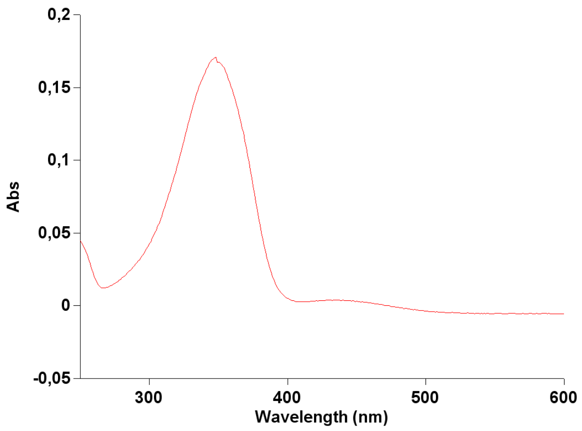

Time (s)	Abs after irradiation
0	0.0045
3600	0.0084
7200	0.0118
10800	0.0156
14400	0.0196
18000	0.0236
21600	0.027
25200	0.0307
28800	0.0339
32400	0.0372
36000	0.0404
39600	0.0433
43200	0.0461
46800	0.049
50400	0.0519
54000	0.055
57600	0.0584
61200	0.0617

3) 30°C UV-vis spectrum before irradiation

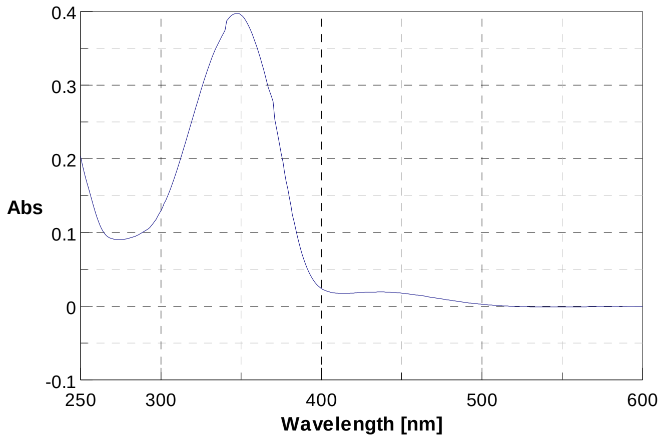

Time (s)	Abs after irradiation
0	0.01755
600	0.01928
1200	0.02054
1800	0.02218
2400	0.02333
3000	0.02444
3600	0.02555
4200	0.02675
4800	0.02796
5400	0.02906
6000	0.03002
6600	0.03115
7200	0.03239
7800	0.0337
8400	0.03494
9000	0.03607
9600	0.03706
10200	0.03814
10800	0.03926
11400	0.04042
12000	0.04157
12600	0.0425
13200	0.04372
13800	0.04463

4) 40°C UV-vis spectrum before irradiation

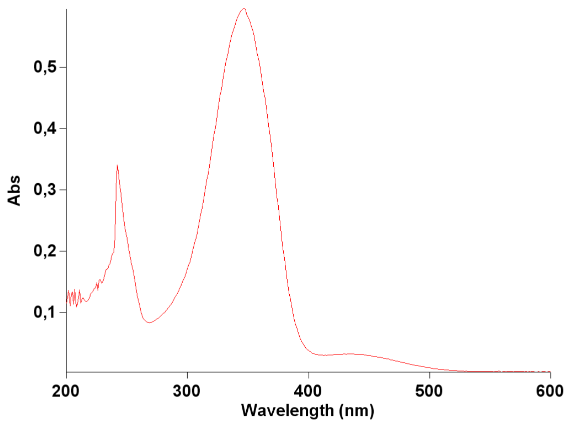

Time (s)	Abs after irradiation
0	0.036
600	0.04293
1200	0.05027
1800	0.05768
2400	0.06457
3000	0.07165
3600	0.07872
4200	0.08542
4800	0.09227
5400	0.09884
6000	0.1057
6600	0.1121
7200	0.1184
7800	0.1248
8400	0.1313
9000	0.1369
9600	0.1434
10200	0.1494
10800	0.155
11400	0.1611
12000	0.1673
12600	0.1726
13200	0.1784
13800	0.1842
14400	0.1895
15000	0.1952
15600	0.2
16200	0.2062
16800	0.2111
17400	0.2168
18000	0.2215
18600	0.2272
19200	0.2319
19800	0.2367
20400	0.2415
21000	0.2468

5) 50°C UV-vis spectrum before irradiation

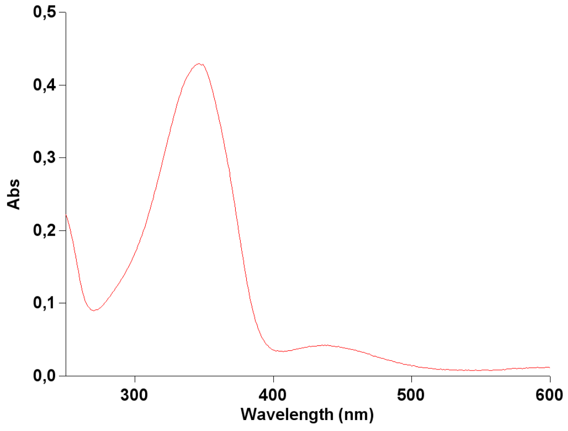

Time (s)	Abs after irradiation
0	0.055
300	0.046
600	0.058
900	0.071
1200	0.082
1500	0.094
1800	0.106
2100	0.1166
2400	0.1279
2700	0.1387
3000	0.1494
3300	0.1597
3600	0.1701
3900	0.1795
4200	0.1896
4500	0.1991
4800	0.2078
5100	0.2186
5400	0.2255
5700	0.2347
6000	0.2424
6300	0.25
6600	0.2589
6900	0.2663
